# Community‐based centers for people living with dementia and family caregivers: implementation and adaptation from pilot to national policy

**DOI:** 10.1002/alz.092500

**Published:** 2025-01-09

**Authors:** Jean Gajardo, Josefina Aubert, Ximena Moreno, Jose M Aravena, Felipe Carcamo, Carolina Barrientos, Maria T Abusleme, Franco Mascayano

**Affiliations:** ^1^ Universidad San Sebastian, Santiago, Santiago Chile; ^2^ Millennium Institute for Care Research (MICARE), Santiago, Santiago Chile; ^3^ Yale University, New Haven, CT USA; ^4^ US Embassy, Santiago, Santiago Chile; ^5^ New York State Psychiatric Institute, new york, NY USA; ^6^ Columbia University, new york, NY USA

## Abstract

**Background:**

Community‐based centers for people living with dementia (PLWD) have been implemented as part of the National Plan of dementia in Chile, delivering a multicomponent and multiprofessional intervention to support the PLWD and their families, integrating social and clinical aspects. We sought to examine the implementation and adaptation of these centers, transitioning from adult day services to a community‐based dementia care model that has escalated to a public policy.

**Method:**

We conducted a realistic evaluation using multiple qualitative data collection strategies and informants to analyze complex interventions, in 4 of a total of 9 centers. Key informants of the centers’ implementation were identified and participated in individual interviews or focus groups, at different levels (macro‐level of centralized policy, n:4; meso‐level of intervention deliverers and center coordinators, n: 27; and micro‐level of user family caregivers, n:14). Interviews and focus groups were audio‐recorded and transcribed. Rapid qualitative analysis was used with predetermined codes describing implementation according to the Active Implementation Frameworks and Implementation Outcomes.

**Result:**

At a macro‐level of implementation, the centers have not experienced relevant transformations, and fidelity is tensioned due to lack of updated centralized technical orientations. At a meso‐level, the centers’ intervention has been adapted to improve sustainability and appropriateness, which has enhanced their scalability. Engagement with the local service network and tailoring according to geographic and cultural context, emerged as implementation drivers. Relevant points of agreement and disagreement regarding implementation among actors were identified.

**Conclusion:**

The implementation of community centers for PLWD in Chile, at a policy macro‐level the program has been continuous and stable. Most relevant implementation drivers and adaptations are identified at a meso‐level, which stresses the need to identify core components and processes for standardization and later evaluation and monitoring. Points of agreement and disagreement among the three levels are key for understanding priorities for improvement and impact.

